# Spirituality and palliative care: international models and new perspectives

**DOI:** 10.3389/fsoc.2025.1523685

**Published:** 2025-07-21

**Authors:** Enrico De Luca, Barbara Sena, Kate Butcher, Lindsay Jane de Wal

**Affiliations:** ^1^Department of Nursing and Midwifery, College of Medicine and Health, University of Birmingham, Edgbaston, Birmingham, United Kingdom; ^2^Department of Health and Care Professions, University of Exeter, Exeter, United Kingdom; ^3^Department of Humanities, Philosophy, Communication, University of Bergamo, Bergamo, Italy; ^4^Oxford Centre for Education and Research in Palliative Care: Sobell House (OxCERPC), Oxford, United Kingdom; ^5^Humanist UK, London, United Kingdom

**Keywords:** spiritual care, end-of-life (EOL), palliative care, spiritual competence, religion

## Abstract

In recent years, healthcare organizations and scholars, particularly in Western societies, have increasingly recognized the importance of the spiritual dimension in patient care. However, this aspect still needs to be fully integrated into everyday practices. Palliative care has begun to emphasize spirituality, addressing patients' psychological and existential needs through a holistic approach beyond the traditional biomedical paradigm. This concept analysis will first explore healthcare and medical professionals' challenges in implementing shared and patient-centered spiritual practices. It will then draw on experiences integrating spirituality in palliative care from Thailand and Italy and introduce two conceptual models for spiritual care and needs assessment from the UK. The discussion will encourage the implementation of integrated models of spiritual needs assessment and care in adult end-of-life and palliative care settings (that address any life-limiting illness). This approach will enable health professionals to effectively address patients' spiritual needs, fostering authentic conversations that are pivotal in integrating models and transforming the care experience into an empowering and meaningful one for staff and patients.

## Introduction

The western biomedical paradigm has considered spirituality and religiosity taboo concepts for decades (Jobin, 2020). This paradigm tended to see physical, mental, and spiritual health as distinct entities, with the body, psyche, and soul being regarded as separate aspects of the human being. Biomedicine can be essentially considered as an “atheistic” or “secular” approach, as it is based on empirical evidence and objectivity. It has created challenges in acknowledging unconventional spiritual healing practices, such as, for example shamanism, Ayurvedic medicine, traditional Chinese medicine, and Tibetan medicine, which have been treated by evidence-based biomedicine as dangerous, unscientific and quacker (Samuel, 2006; Tian et al., 2021). However, there is a growing contemporary discourse on the evidence base studies on Chinese and Ayurvedic Medicine that are considered by the World Health Organization (WHO) and academic world (Hoenders et al., 2024). At the same time, Western biomedical approach tends to increasingly exclude religious considerations from bioethical issues, such as abortion, end-of-life management, sexual reassignment, or sterilization interventions. However, in contexts still strongly guided by a religious culture, such as Christian, Jewish, Muslim, medical practice still seems to contrast the atheistic paradigm imposed by the biomedical approach (Balboni and Balboni, 2011). In fact, it is still possible to witness the application of rules, procedures, and medical protocols in the management of ethically and religiously sensitive clinical events (e.g., sterility, pregnancy, the prevention of certain diseases, euthanasia, etc.) which, although considered as “conventional,” nevertheless contrast strongly with those adopted in more secular contexts and culturally free from religious influences (Cherry, 2019).

Another critical aspect is that spiritual wellbeing is often believed to be synonymous with psychological wellbeing. Although the two concepts are undoubtedly interrelated, they refer to different dimensions of the human experience. While psychological wellbeing aims to improve the health of the psyche by treating and preventing mental illnesses, spiritual wellbeing focuses on the inner existential dimension of the individual (Fisher, 2011). The incorporation of spirituality into healthcare practices is hindered by the complexity and overlapping of spiritual concepts, making it difficult to form a shared, formalized definition of spirituality. This has also hindered its recognition among medical and healthcare workers practice (Puchalski et al., 2020; Jones et al., 2021; Rykkje et al., 2022), and the inevitable consequence is a “marginalization” of the religious and spiritual dimensions from daily healthcare practice.

From this general observation, the relationship between medicine and spirituality, as well as the connection between science and religion, is a complex subject that is not easy to address, explain, or incorporate into clinical and healthcare practices. These relationships can be challenging to integrate into the various treatment and pathways approaches. This also explains the fact that the WHO has so far failed to include the spiritual dimension in its definition of health, despite several attempts since the beginning of the 1980s, especially driven by Islamic countries and the Mediterranean area (Chirico, 2016). For example, WHO has a policy on Traditional Medicine since the early part of this millennium which addresses spiritual aspects of health but not speaking to biomedical health and connect with spirituality (World Health Organization, 2013). The only area of Western medicine in which spirituality has begun to show itself more as a relevant element in patient treatment seems to be palliative care and, more generally, end-of-life management. These fields represent contexts of care at the fringes of traditional western biomedical paradigm. The classic clinical mindset, in fact, tends to consider and valorise only active and pragmatic attitudes toward the disease and the patient, i.e., aimed exclusively at their recovery or at least survival (Freidson, 1988, p. 168 et seq.). When biomedicine can no longer be effective—such as in cases involving dying patients or those with life-limiting conditions—the medical profession recognizes a “failure” and cedes control over care models. This shift paves the way for alternative approaches, including non-conventional or holistic methods. These approaches are becoming more prevalent and signify a substantial evolution in palliative care (Gawande, 2014; Sena and De Luca, 2022). However, although the dimension of spirituality has been progressively recognized and included considerably more in this area than in other medical-healthcare practices (Saunders, 1967; Best et al., 2023), there is still a lack of a shared definition of spirituality and spiritual care practices. There is criticism, in some texts, that spiritual care is conceived in a Westernized, monotheistic, manner thus demonstrating an irrelevance to non-western communities, and not recognizing that religious beliefs are only a part of a person's spiritual beliefs (Lundberg et al., 2024). In addition, palliative care staff often lack specific training in addressing spiritual needs, relying instead on spiritual assistants, who are not involved in clinical care, to support dying patients from a spiritual or religious perspective (Batstone et al., 2020; Chahrour et al., 2021). However, spirituality can serve as an innovative and alternative approach to the reductionist and empiricist practices typical of the biomedical model in these care settings (de Diego-Cordero et al., 2022).

This paper aims to provide a conceptual analysis of the relationship between spirituality and palliative care, emphasizing the increasing attention that the issue of spirituality has received in recent years in healthcare context, specifically in end-of-life and palliative care domains. Therefore, the primary aim of this paper is to carefully study, clarify and critically analyse the concept of “spirituality in palliative care” through a detailed understanding and evaluation of the term (Foley and Davis, 2017; Smith and Mörelius, 2021). Its purpose is not to provide a systematic literature review or a scoping review but to provide an interpretive analysis of innovative theoretical and empirical models that support, clarify, and expand the definition of spirituality in palliative care.

The paper's first section will explore the concepts of religiosity and spirituality and their relationship to palliative care. This introduction will reference definitions from various international health organizations to provide a clear context for our analysis. Next, the paper will focus on healthcare professionals' applications of spiritual care while highlighting existing gaps in this area. Finally, it will present and discuss international models of spirituality in palliative care that have not yet been widely adopted in health services. These models represent pioneering and innovative efforts to address the limitations of the current biomedical approach to spiritual care, which emphasizes a quantitative assessment of symptoms. This emphasis risks depersonalizing care and overlooking individuals lived experiences of illness.

## Beyond religiosity

Considering the wide-ranging and somewhat ambiguous interpretations of spirituality, it is important to clarify, although not exhaustively or definitively, what this concept means in relation to religiosity within the healthcare sector, particularly in palliative care. Puchalski et al. (2014) define the spiritual as a dynamic and intrinsic aspect of humanity whereby individuals seek to find meaning, purpose and transcendence as a relational experience with themselves, others and the community. Spirituality, with its elements of subjectivity, is an integral part of each person's life and individual sphere. It is related to a variety of cultural, historical and social factors, as well as influenced by beliefs, values, traditions, personal convictions and practices. It can, therefore, take on different meanings depending on the circumstances and must be adapted to the personal and cultural context of reference (Murgia et al., 2020; Puchalski et al., 2014). For this reason, spirituality must be considered a complex, diversified, and nuanced phenomenon, and rich in its diversity of experiences, which everyone can undertake in every area of their life in all possible spiritual and religious inclinations. In contrast, religiosity refers to a concrete set of beliefs, practices, and languages that characterize a community that seeks transcendent meaning in particular ways, based on faith in a deity but also interpersonal and institutional commitment with a formal religious group with its doctrines and traditions (Arrey et al., 2016). The term “religiosity” refers to a set of beliefs and practices within a specific tradition and community. Spirituality is a more inclusive term that encompasses a person's connection to a larger reality, giving life meaning. Spirituality can range from appreciation of nature to the hope for immortality through scientific discovery. Therefore, anyone can have their own spiritual dimension without adhering to any specific religion (Peteet and D'Ambra, 2011).

In recent years, there has been growing interest in spirituality across different fields and activities, reflecting cultural, religious, and societal changes. This trend, especially prominent in Western countries, signifies a shift from institutionalized religion to a more personal quest for meaning and self-knowledge. While regular church attendance, religious belief, and the influence of religious institutions in daily life are declining in Western society, belief without affiliation to any specific religion is growing, transcending race and ethnicity. This has led to a separation between religiosity and spirituality, with an increasing emphasis on the latter over the former (Nita, 2019).

Across several healthcare settings (e.g., oncology, palliative care, emergency departments during pandemic, critical care, care of the older person, migrant health) there is an increasing focus on spirituality as a tool to support patients with various medical conditions to improve person centered care (Long et al., 2024). Spirituality has been recognized as an essential resource for improving psychological health and developing positive attitudes toward healing from illnesses (Timmins and Caldeira, 2017a; Jaberi et al., 2019; Zhang et al., 2024). Furthermore, spiritual health can help regulate stress by promoting coping strategies that positively impact health behaviors. Patients whose spiritual needs are acknowledged from the beginning of treatment report greater satisfaction with their care (Hubbell et al., 2017; Lundberg et al., 2024). Many studies have explored the benefits of spiritual care for patients at the end-of-life (Best et al., 2023). Recognizing the need for spiritual support as part of a patient's overall treatment process, especially in palliative care, has gained recognition and is now included in various international, national, professional guidelines and documents. For instance, WHO defines palliative care as aiming to improve the patient's quality of life and alleviate their physical, psychological, social, and spiritual suffering (World Health Organization, 2020). Guidance from NHS Golden Jubilee (2023) states areas for specific staff education to include the visibility of spiritual care, bereavement and grief, and holistic care.

In 2010, the European Association for Palliative Care (EAPC) established a group of experts from various health and medical professions to explore and define the spiritual dimension of palliative care. This group produced a *white paper on multi-disciplinary education for spiritual care in palliative care* to support staff in introducing spirituality in end-of-life care (Best et al., 2020). The EAPC white paper emphasizes that a higher level of personal spirituality and competence in spiritual care are linked to reduced burnout among palliative care professionals. It also points out that paying attention to a patient's spiritual needs is still a relatively new concept in many healthcare settings. Therefore, healthcare and medical staff must practice personal spiritual reflections as a professional requirement. To address this, the white paper recommends providing universal training in spiritual care for all palliative care staff and developing a Multidisciplinary Model of Spiritual Care tailored to this specific context. Additionally, fostering spiritual care education and encouraging a self-reflective approach to personal spirituality can help alleviate a caregiver's lack of confidence in providing spiritual care and positively impact the spiritual distress they may experience (Timmins and Neill, 2013; O'Brien et al., 2019). Other guidance is emerging regarding formal multidisciplinary education for staff, such as updated certification from Spiritual Care Association (2025) and local organizational training e.g., NHS Golden Jubilee hospital in Scotland (NHS Golden Jubilee, 2023).

In the United Kingdom, for example, healthcare services have developed various frameworks at national and local levels to support the culture of palliative care, in which attention to the spiritual dimension is increasingly included. These documents reinforce the idea that the experience of suffering associated with physical symptoms at the end-of-life can be exacerbated, or sometimes caused, by emotional or psychological distress or social or spiritual distress. Therefore, professionals are encouraged to work increasingly on developing individualized care plans that include explicit consideration of the person's comfort, symptom control, and psychological, social, and spiritual support (NHS, 2021).

In hospitals and healthcare settings that focus on end-of-life care, such as hospices, specific non-healthcare professionals are traditionally assigned to provide spiritual care. These roles typically include chaplains or religious representatives, as well as secular figures like non-denominational spiritual assistants. These individuals are sometimes part of the multidisciplinary palliative care team. In several countries, including Italy, their presence is becoming more common, but challenges related to integration and professional recognition remain (Kruizinga et al., 2016).

Anke et al. (2019) suggest that the involvement of these specific non-healthcare figures in the spiritual care of terminally ill patients is increasingly questioned. Numerous studies indicate that all members of the care team—including doctors, nurses, psychologists, and social workers— should be capable of addressing patients' spiritual needs. Healthcare professionals can play a vital role in identifying and alleviating spiritual distress (Best et al., 2023). Furthermore, Meeting the spiritual needs of patients is also considered essential for reducing burnout among healthcare staff working in high-intensity care environments (Rushton et al., 2015). Healthcare professionals, particularly in palliative care, often grapple with existential questions and emotional challenges, even with their preparation for dealing with death and dying (Best et al., 2020). Spiritual care is a crucial element of healthcare, especially in nursing, which emphasizes building strong relationships with patients (Hsiao et al., 2010). Nursing profession has traditionally emphasized the spiritual aspect of care, reflecting its importance in patient treatment. To support this focus, guidelines and codes of conduct have been established that influence nursing practices globally. The International Council of Nurses (ICN), in its most recent *Code of Ethics for Nurses*, which has served as a global standard since 1953, reaffirms that “the nurse promotes an environment in which the rights, human values, habits, religious, and spiritual beliefs of the individual, families, and communities are recognized and respected by everyone” (International Council of Nurses, 2021, p. 7). This acknowledgment of the significance of spiritual care places substantial responsibility on nursing professionals, educators, and policymakers in healthcare. In response to the ICN's request, the regulatory nursing councils in Italy (OPI) and the United Kingdom (NMC) have incorporated the spiritual dimension of person-centered care into their ethical codes as a reflection of a holistic approach. Specifically, regarding end-of-life care, the Italian nursing council emphasizes “the importance of the caring gesture, shared care planning, palliation, and providing environmental, physical, psychological, relational, and spiritual comfort” [Federazione Nazionale Ordini Professioni Infermieristiche (FNOPI), 2025, art. 27 code of ethics]. The NMC reiterates the importance of assessing the person's spiritual needs to provide effective holistic care in the recent campaign “Seeing the whole person supports better care” (Nursing Midwifery Council, 2024). Thus, nurses must carry out a needs assessment and care planning according to the patient's mental, physical, cognitive, behavioral, social and spiritual needs, making decisions with the person and not for the person. Interestingly, alongside the explicit interest in spirituality from nursing professional associations, much of the scientific literature on spirituality in care is found in nursing journals, though infrequently in those originating from Southern European and African settings (Matos et al., 2024). However, nurses, particularly in Western healthcare settings, often do not provide explicit spiritual care to their patients. Many still need to conduct initial spiritual screening assessments (Taylor et al., 2017). In practice, nurses frequently rely on their own religious beliefs and cultural affiliations to support both patients and their families. This approach often serves as a coping mechanism for the daily demands and psychological stress of their work (McLouth et al., 2021; Cruz et al., 2022). In other words, belief in a higher power or faith in God often becomes an integral part of the healthcare professional's personal approach to their work in many healthcare contexts (Davoodvand et al., 2017). The lack of adequate training in practicing spiritual patient care is one of today's main obstacles. To try to fill this gap, some countries, such as the United States, Germany and the Netherlands, have begun to include spiritual care in medical and nursing training courses, albeit in a generic and non-prescriptive form (Nissen et al., 2021). The training programs for providing spiritual care can vary significantly from one another. This is because offering this type of care in a diverse healthcare context is complex, as it requires consideration of a wide range of patients' secular, spiritual, and religious beliefs.

Even in terms of spiritual care “practices,” there are no standardized or widely accepted models among medical and health professionals (Jones et al., 2021). The tools used for this type of intervention, such as questionnaires or interviews to assess spiritual needs, are usually created independently by the healthcare professional and do not form a cohesive approach in the patient's treatment. In other words, spiritual needs are viewed as individual and dependent on the relationship between the medical or healthcare professional and the patient. As a result, spiritual care is can be seen as something improvised, based solely on the professional's experience and personal sensitivity, and can be inconsistently implemented or ignored altogether (Austin et al., 2018).

## Differentiating models and dimensions of spiritual care

Spiritual care in palliative contexts is not a singular practice but exists along a spectrum of models shaped by cultural, institutional and philosophical frameworks. A growing body of literature distinguishes between three main types: clinical, social and holistic spiritual care models—each with distinct assumptions, actors and methods (Table 1).

**Table 1:** Models of spiritual care.

**Type of model**	**Definition**
Clinical spiritual care model	The clinical model operates within institutional healthcare environments and is usually delivered by professionally trained providers such as chaplains, nurses, or psychologists. These interventions are typically structured, evidence-based, and aligned with broader interprofessional care plans. Spiritual assessments like FICA or HOPE (Anandarajah and Hight, 2001) exemplify this model's systematic approach. Recent studies confirm that spiritual competence among clinical staff contributes to improved outcomes and reduced caregiver burnout (Mascio et al., 2024; Costeira et al., 2024). However, its reliance on formal tools can risk medicalizing or depersonalizing spiritual encounters.
Social spiritual care model	By contrast, the social model is relational and community-oriented, often practiced outside formal medical institutions. It involves family, faith or belief groups, volunteers and community leaders who provide emotional, moral and existential support. Rumbold (2012b) emphasizes that such care is grounded in solidarity, shared meaning, and social rituals, rather than professional expertise. This model is especially relevant in culturally diverse or resource-limited settings where communal care traditions are strong.
Holistic spiritual care model	The holistic model integrates elements of complementary medicine, traditional spiritual practices and trans-religious philosophies. It recognizes that spirituality may be expressed through connection to nature, art, mindfulness, ancestral rituals, or energy-based healing.

These distinctions clarify the evolving landscape of spiritual care and allow healthcare teams to better match models with patient values, cultural contexts as well as care settings.

Balboni and Balboni's (2018) work *Hostility to Hospitality* offers a powerful critique of biomedicine's neglect of spiritual care and champions the inclusion of religious meaning-making. While their insights have helped institutionalize spiritual care in clinical settings, their framework remains strongly anchored in faith-based traditions. As such, their framework may fall short in accounting for secular spiritualities, existential pluralism, or non-religious worldviews increasingly present in today's healthcare environments. Therefore, a contemporary approach must move beyond the religious/non-religious binary to encompass a broader landscape of meaning.

### Trans-professionalism, secularization, and philosophical grounding

Spiritual care is inherently trans-professional: it cuts across disciplines and challenges hierarchical boundaries. Nurses, physicians, chaplains, social workers and volunteers all encounter spiritual needs in practice, and each brings different perspectives and competencies to this work (Best et al., 2023). Recognizing spiritual care as a trans-professional domain encourages collaboration, dialogue, and mutual respect across fields. Professionalizing spiritual care does not require stripping it of depth or sacredness. Instead, it invites internal differentiation—a framework where religious chaplains maintain their faith-based roles, while others—such as non-religious-based chaplains—provide existential-spiritual or psychosocial care.

This pluralistic approach respects both theological specificity and humanistic diversity (Doehring, 2024; Van Dijk, 2021). It also reflects real-world demographics, where many patients identify as spiritual-but-not-religious, atheist, agnostic, or culturally affiliated without doctrinal belief.

Spiritual care must also be understood within the lineage of philosophy as a form of spiritual practice. Traditions such as Stoicism, existentialism and even contemporary eco-philosophies provide tools for cultivating resilience, meaning and ethical reflection (Zinevych, 2018). Integrating philosophical exercises—such as journaling, values clarification, or dialogical inquiry—into care settings can enrich the depth and inclusivity of spiritual support. Healthcare providers trained in reflective practice may be better equipped to accompany patients through existential uncertainty.

By framing spiritual care through clinical, social, and holistic lenses and grounding it in trans-professional, pluralistic and philosophical perspectives, healthcare systems can better address the full spectrum of patient needs at the end-of-life. This nuanced approach not only fosters inclusivity but strengthens the ethical and relational core of palliative care (Table 2).

**Table 2:** Comparison of spiritual care models.

**Model**	**Core characteristics**	**Primary providers**	**Context/setting**	**Limitations**
Clinical model	Evidence-based, institutional, integrates assessments (e.g., FICA, HOPE), outcome-focused	Trained professionals (e.g., chaplains, nurses, physicians)	Hospitals, hospices, formal palliative care settings	May medicalise spirituality or overlook non-religious expressions
Social model	Community-driven, relational, emphasizes solidarity and shared meaning	Families, communities, volunteers, faith groups	Home care, community, informal support networks	Lacks standardization; limited in clinical integration
Holistic model	Trans-religious, includes traditional healing, nature connection, philosophical elements	Traditional healers, culturally trained practitioners, spiritually attuned staff	Culturally diverse settings, integrative and alternative medicine environments	May lack empirical validation; variable professional legitimacy

### International models of spiritual care in palliative care settings

Models of spiritual care are often context-based and given the lack of widely embraced models among palliative care teams in Europe and the Western world, it is important to explore other countries approaches. Reflecting on established practices from regions where allopathic, biomedically informed medicine coexists with other healing systems, such as in Asian countries, can lead to thoughtful consideration and practical solutions. Concepts and practices from Italy and the UK can offer different perspectives inspired by Asian philosophy or western non-religious spiritual care models.

#### Thailand and Italy: spiritual care before physical

In the introduction, it was mentioned that in Asia, various treatment approaches integrating the spiritual dimension of healthcare professionals who work with palliative care patients are more common compared to other continents. Among the end-of-life care models, particularly in the Buddhist tradition in Asia, spirituality plays a significant role. In Thailand, healthcare professionals in palliative care have developed different aspects of spirituality aligned with holistic nursing practices that focus on caring for the whole person (Sukcharoen et al., 2020). They emphasize the importance of integrating spirituality into the practice of caring for oneself and others, making it a practical part of their daily life and profession. Recent ethnographic studies conducted in the Thai context have found that spiritual care is deeply integrated into the daily routine of a palliative care center (Davis et al., 2023; Upasen et al., 2022). The medical and healthcare staff of the center, where the study was conducted, reported that Buddhist principles play a significant role in providing meaning and offering solutions to moral dilemmas and stress in palliative care. The study revealed that spiritual aspects are integrated into daily practices, guiding actions, reflections, and the way patients and professional issues are handled. The entire community, including patients, family members, caregivers, and staff, actively participate in daily hospice care and spiritual activities. They engage in collective spiritual support through activities such as meditation, singing, several types of massages, and other shared practices (De Luca et al., 2022). These studies have also hypothesized that palliative care staff can effectively alleviate the challenges of a strict biomedical approach to spirituality by adopting a spiritual perspective on death and dying, viewing it as a natural part of life (Buddhaghosacariya, 2019).

In Italy, similar experiences are subject to experimentation with new palliative care pathways. For example, the hospice born within the “*Borgo Tutto è Vita*” project represents a unique example that addresses the theme of spirituality, placing it at the center of the care and activities of the entire structure (Borgo Tutto è Vita, 2023). The center, born from the long reconstruction of an abandoned village in Tuscany (Prato), is focused on the accompaniment toward death according to a project where spirituality becomes an effective model of care. As for the Thai center, end-of-life care becomes an opportunity to reflect on the meaning of life and accompany the person on a path where, among the fundamental palliative needs (nutrition, alleviating symptoms, decreasing anguish), the spiritual is considered an integral part of care plans. In the *Borgo*, the operational modality in accompanying death is expressly non-religious; therefore, it is respectful of the sensitivity and beliefs of the person and aiming to support their internal resources. The center provides palliative care from the early stages of diagnosis for illnesses with potentially terminal outcomes and has significantly impacted as an innovative alternative to the traditional hospices in Italy and is now formally recognized and supported by the National Health Service in Tuscany (SSN, Toscana). It is a place where several spiritual care activities are central and valorised, while healthcare is still provided according to the best clinical standards of biomedical approach. In particular, the approach proposed considers each aspect of healthcare (body, psyche, relationship) integrated with the spiritual dimension. Therefore, every therapeutic action is planned in a spiritually focused mode, influencing all the other interventions. The *Borgo* employs a team of health professionals with extensive experience in spiritual practice and meditation. Spiritual care activities are incorporated and adapted into normal daily activities. For example, professionals and volunteers attend regular group meditation appointments and ordinary team meetings. Like the Thai hospice, the facility includes a residential structure within the village to accommodate patients with incurable diseases, as well as their family members who may stay even after the patient's death. This approach integrates spiritual care at various levels and moments in a personalized and organic way, involving the entire community, like the experience in Thailand.

These two brief examples illustrate how end-of-life care is conceptualized and offered when it is centered on the individual's needs. This approach differs from the traditional biomedical perspective, which often prioritizes healing at all costs (Bueno and La Calle, 2020). Instead, the focus shifts to identifying and addressing people's needs—recognizing that these needs are not just clinical but also existential in nature. Therefore, placing the spiritual dimension at the center of the care model means incorporating it into the care plan. This involves a thorough assessment of the individual's spiritual needs. The palliative care team is responsible for developing a structured and personalized plan that is explicitly shared and supported by all professionals involved.

#### UK models: fellow traveler and horizontal transcendence

Palliative care in the UK is offered in all healthcare and domiciliary settings by the whole multidisciplinary team—in primary, secondary and tertiary care settings. It is perceived to be a suitable and relevant aspect of generalist care for all health and social care practitioners to undertake, and this includes spiritual care. Several assessment and intervention tools intended for end-of-life and palliative care may be identified within the literature (Timmins and Caldeira, 2017b). However, there is no one example of a validated tool or evaluated model (developed specifically for palliative and end-of-life care) which is in common use in clinical or other UK practice settings. The models discussed in this section are rarely implemented by generalist staff, however many staff would agree that they do offer spiritual care as a part of their daily role.

A critical aspect of integrating spirituality into palliative care involves the role of specialized professionals known as chaplains. Traditionally rooted in Christian practices, NHS chaplaincy services in UK have expanded to include multi-faith and belief teams, reflecting patients' increasingly diverse religious and non-religious demographics (Van Dijk, 2021). The NHS chaplaincy guidelines explicitly stated that chaplaincy services are no longer affiliated with any one religion or belief system, thus promoting a more inclusive approach to spiritual and pastoral care (Swift, 2015). This shift has been driven by the recognition that a significant portion of the population identifies as non-religious and requires pastoral care that aligns with their secular worldviews (Humanists UK, 2017). Integrating humanist chaplains into traditionally faith-based teams is a significant development in ensuring that pastoral care is accessible and relevant to all patients, regardless of their religious or non-religious backgrounds (Orton, 2008). Humanist chaplains focus on meaning-making, purpose, and existential questions without relying on religious doctrines, providing crucial support to non-religious patients (Savage, 2015).

However, supporting people during the difficult journey into end-of-life demands many skills from health practitioners. Fopka-Kowalczyk et al. (2023, p.2012) describes them as a need for “a fellow human being capable of showing concern, recognizing emotions, talking, and offering help.” A common description by staff would be that they are “being with” or “journeying with” a patient. The “Fellow Traveler Model” promoted by Holloway and Moss (2010), a social worker, incorporates aspects of narrative and biographical approaches, along with a self-awareness within the practitioner. According to literature on the topic, the model takes a “common humanity” approach to spiritual care (Universities of Hull, Staffordshire and Aberdeen, 2011). The Fellow Traveler model is one of relational understanding as opposed to transactional operating, as often occurs when healthcare staff are working in busy, time-pressured the model emphasizes the need to “be a companion,” helping the journey along, who listens and shares, guides and sustains. Sweeney et al. (2009) describes it thus, “One's guides in this world have a dual role: to read the map and direct you accordingly, but also to be with you on the terrain, a place of great uncertainty” (2009, p. 122).

The relevance of this model is that practitioners can join and leave the journey at many points, supporting as they feel able but passing over the care if they feel a lack of competence (Table 3). The Fellow Traveler model adds a rich means of understanding and articulation of the care that we offer at the end-of-life. Such care is relational in nature, holistic and demands self-awareness on the part of the practitioner. Knowing that we are given permission to end our personal journey with a patient at a time when we feel “out of our depth” and so hand over to a better-qualified practitioner such as a chaplain gives a degree of personal emotional safety to all involved. Thus, the model is appropriate for all professionals and levels in the multidisciplinary team. It is worth mentioning that “Spiritual care takes places within genuine human encounters” (Rumbold, 2012a, p. 182) and so narratives, or life stories, are a common way for patients to articulate their spiritual needs and support mechanisms. Therefore, a model that encourages narrative aspects of care could be more indicated for spiritual care in end-of-life.

**Table 3:** Fellow Traveler Model—how to use, in Holloway and Moss (2010).

**Levels**	**Definition**
Level 1-“*Joining*”	Health or social care workers engages with an individual. This requires a degree of spiritual awareness in themselves as well as in others (an ability to identify people who may have a spiritual concern). The professional is starting with where the person is on their spiritual journey. All members of the team should have the competence to undertake this, to ‘hear' what the person is saying and identify cues.
Level 2-“*Listening*”	It requires spiritual sensitivity. Using level 1 skills, the practitioner develops their skills to undertake a preliminary assessment of the nature and significance of the spiritual need. At this point, joint working with another member of the multidisciplinary team may be established, or the practitioner may feel able to continue to be the traveler with the individual.
Level 3-“*Understanding*.”	This requires spiritual empathy, and an understanding of how spiritual resources may be used. Practitioners need a degree of self-awareness to be able to effectively develop the relationship further, and at this point may choose to end their companionship and ask a suitable colleague to share the journey further.
Level 4-“*Interpreting*”	Using the metaphor of a journey, this may be a mountainous, or rocky, area where the need for a guide is clear. Through choosing to travel with the individual, a practitioner often needs to make themselves vulnerable here; to utilize tools such as meaning making or an ability to engage with hope (or hopelessness) this is deeply involved work requiring care and persistence and so is often allocated to a professional spiritual care provider.

As the integration of spirituality into palliative care continues to evolve, there is a pressing need to widen and educate staff around spiritual assessments. This effort must also debunk the myth that spirituality is solely reserved for those of faith or solely left to the chaplain's responsibility. Spiritual assessments should encompass a broader, existential dimension that addresses the spiritual needs of all patients, regardless of their religious beliefs or lack thereof. Spiritual assessments in palliative care are crucial for understanding and addressing patients' diverse needs (Timmins and Caldeira, 2017b). These assessments help healthcare providers identify sources of spiritual distress and provide right interventions to support the patient's overall wellbeing. Therefore, widening the scope of spiritual assessments in palliative care to include existential dimensions is essential for providing comprehensive, patient-centered care.

Horizontal Transcendence is a model in use by chaplaincy which health professions are also encouraged to apply in their contexts (Streib and Hood, 2013). This model encompasses a feeling of awe, respect, and gratitude for the mysteries of life, without the need to reference a higher power or deity. Horizontal Transcendence, within the framework of existential anthropology, highlights the integration of personal existential, and universal spiritual aspects in human character. This concept introduces a trans-empirical cultural realm that encompasses human senses, images, and symbols. It posits that being human is an ongoing endeavor, where individuals move beyond their own personal identity toward the “super-individual” by becoming part of a spiritual community or a universal cultural experience (Zinevych, 2018). The concept of Horizontal Transcendence emphasizes interconnectedness between individuals, communities, and the environment, providing a more inclusive approach to spirituality that resonates with religious and non-religious individuals. This perspective is crucial in palliative care to address the holistic needs of patients. Horizontal Transcendence involves finding meaning and connection through relationships, community engagement, and interaction with nature. It recognizes that spiritual fulfillment can be achieved through diverse avenues beyond traditional religious practices. Interpersonal relationships, community engagement, and connection with nature are core components of Horizontal Transcendence, contributing to a sense of purpose, wellbeing, and spiritual fulfillment. Therefore, Horizontal Transcendence challenges the traditional boundaries of spirituality by emphasizing the significance of non-religious sources of spiritual fulfillment. This broader perspective has important implications for spiritual care and wellbeing. By recognizing the diverse ways individuals can achieve spiritual fulfillment, practitioners can adopt a more inclusive approach to spiritual care that respects and values different paths to spirituality. Recognizing and embracing this form of transcendence can lead to more inclusive and holistic approaches to spiritual care and wellbeing which is not solely fixed on whether a patient has a faith.

Spiritual assessment tools provide structured approaches to exploring patients' spiritual needs, thus help clinicians engage in meaningful conversations with patients about their spiritual concerns and integrate this information into their care plans. Instruments such as the FICA Spiritual History Tool (Faith, Importance, Community, Address in Care) and the HOPE Questions (sources of Hope, Organized religion, Personal spirituality and practices, Effects on medical care and end-of-life issues) provide structured approaches to exploring patients' organizations (Sweeney et al., 2009). In contrast to a professional who “does” for the person, spiritual needs (Anandarajah and Hight, 2001). Both the FICA and HOPE tools underscore the importance of integrating spiritual assessments into healthcare. They provide structured ways for healthcare providers to engage with patients about their spiritual needs, which can be critical for comprehensive care. The Horizontal Transcendence model with its specific questioning style can be used and integrated by healthcare staff working in palliative or end-of life care (Table 4). The Horizontal Transcendence model can offer an integration to these popular tools by expanding them to include questions on about relationships and community (Table 5).

**Table 4:** Assessment through conversations (de Wal, 2024—inspired by Horizontal Transcendence Model).

**Active listening: Engage in deep, active listening during patient interactions**.	**This involves being fully present, showing empathy, and allowing patients to share their stories and experiences without interruption**.
Open-ended questions: Often closed questions upon intake are utilized in healthcare where someone is asked for their ‘religion'.	This question already implies someone has a religion, rather than actively asking the question what someone's worldview might include. Use therefore open-ended questions to explore the patient's sources of meaning and connection. Examples include: “Can you tell me about the people who are important in your life?”; “What activities or places bring you a sense of peace and fulfillment?”; “How do you stay connected with your community or loved ones?”
Reflective responses: Provide reflective responses that confirm the patient's experiences and encourage further sharing.	This helps build trust and a deeper understanding of the patient's spiritual needs.

**Table 5:** Exploring relationships and community (de Wal, 2024—Inspired by Horizontal Transcendence Model).

**Areas**	**Examples**
Family and friends	Assess the quality and significance of the patient's relationships. Ask about their interactions, the support they receive, and how these relationships contribute to their sense of identity and purpose.
Community engagement	Inquire about the patient's involvement in community activities, clubs, or volunteer work. Understanding their role in the community can reveal important aspects of their spiritual life.
Support networks	Identify key members of the patient's support network and their impact on the patient's well-being. Encourage the involvement of these individuals in the patient's care plan.
Connection with nature	Nature-based questions: Ask patients about their relationship with nature and how it influences their spiritual well-being. Questions can include: “Do you find comfort in being outdoors or engaging with nature?”; “Are there particular natural places that hold special meaning for you?”. Facilitating access: Where possible, facilitate access to natural settings or bring elements of nature into the patient's environment. This could involve creating opportunities for patients to spend time in gardens, organizing nature walks, or incorporating natural elements into their living spaces.
Personal values and existential concerns	Value-based questions: Explore the patient's core values and how these shape their life choices and sense of purpose. Questions can include: “What values are most important to you, and how do they guide your life?”; “Are there particular beliefs or principles that help you find meaning in difficult times?”
Existential exploration	Address existential concerns by discussing the patient's thoughts on life, death, and legacy. This can help patients articulate their spiritual needs and find peace.

For example, we can add questions to the FICA and HOPE tools to ask about connections with nature and community involvement in the broadest sense of community (e.g., not solely reserved for religious communities). The question around “Faith” or belief within FICA, or the “O” for organized religion in the HOPE tool, should be counter-balanced with asking about one's worldview in an open manner. This can be asked in a way that opens a conversation, such as “Do you hold any religious or non-religious beliefs,” or “What beliefs sustain you when things get tough?”.

These examples are essential because they show how the evolution of palliative care from a religious-based approach to spiritual care can be supported by engaging with and transforming the assessment tools and tailoring them to each person's needs.

## Discussion

This conceptual analysis presented various models and suggestions for implementing spiritual care in palliative and end-of-life scenarios, raising essential questions about how to review and enhance care for this vital aspect of the human experience of illness and dying. The models from international experiences places spirituality at the center as a guiding element in the clinical path, supporting, however, the rigorous and evidence-based background of traditional medicine, which is neither denied nor marginalized, but on the contrary, it becomes “embedded” within a holistic and patient-centered approach. Thus, spirituality should be considered alongside other dimensions included in the care plan, such as pain management, nutrition, symptom relief, and overall comfort (Timmins and Caldeira, 2017a).

All this, however, requires an important, and in some cases radical, cultural change for medical and healthcare professionals, which cannot be improvised but needs to be supported at an institutional, healthcare, and educational level. Incorporating spiritual care education into medical and nursing curricula can enhance healthcare providers' ability to offer holistic care (Puchalski et al., 2020). Educational initiatives are needed to equip healthcare professionals with the skills and knowledge to conduct comprehensive spiritual assessments. Training programs should emphasize the distinction between spirituality and religiosity and promote an understanding of how to address the spiritual needs of all patients. By educating healthcare professionals and debunking myths about spirituality, we can ensure that all patients receive the spiritual support they need. This approach not only enhances the quality of care but also aligns with the holistic philosophy of palliative care, ultimately improving the wellbeing of patients at the end-of-life. It is essential to recognize that spirituality can include existential concerns, personal values, and connections with others and the world around us (Puchalski et al., 2014).

This paper emphasizes also the need for medical and healthcare staff to broaden their perspectives on end-of-life care by incorporating spirituality and being open to integrated assessment approaches to achieve a more holistic person-centered care. The integration of methodologies should occur once standardized spiritual assessment tools—such as scales and scores—are combined with narratives and person-centered approaches, as briefly illustrated in the Horizontal Transcendence and Fellow Traveler models. Interestingly, experiences from the UK—where palliative care has evolved significantly since Cicely Saunders' pioneering work and is now well-embedded within the NHS—show that healthcare professionals still seek standardized tools to guide care delivery, even in less biomedical-focused environments like palliative and end-of-life care settings. Furthermore, while the biomedical approach is crucial in many contexts, end-of-life care requires a comprehensive and humanistic perspective (Kaasa et al., 2018). Research indicates that this approach can also assist health professionals in finding meaning when faced with the profound experience of death and dying (Best et al., 2023). Enhancing the dialogue between narrative and numerical approaches is critical for delivering authentic spiritual care. Although achieving this balance can be challenging, it can lead to a comprehensive understanding of patients' needs and reassure healthcare staff of their ability to provide effective care. Traditionally, healthcare professionals have been socialized into viewing spirituality and religion as services provided solely by chaplains, without actively engaging in the process themselves (Chahrour et al., 2021). It is time to redefine this position and take a more inclusive approach to spiritual care. It is essential for medical and healthcare professionals to develop comprehensive models that integrate body, mind, and spirit, ensuring that care is personalized according to the patient's authentic needs, rather than merely clinical or pharmacological considerations.

## Conclusion

Addressing the spiritual needs of patients at the end-of-life leads to many positive outcomes for patients, their families, and healthcare professionals. Palliative care staff can start conversations about spirituality by using authentic communication and incorporating validated international spiritual assessment tools. Enhancing spiritual care at the end-of-life requires providing training and support for staff, which not only benefits patients but also empowers staff to offer improved support. Furthermore, effective spiritual support in end-of-life care needs a paradigm shift among medical and healthcare staff, allowing them to feel empowered, justified, and authorized to provide this fundamental aspect of care.
